# TTV and HPV co-infection in cervical smears of patients with cervical lesions

**DOI:** 10.1186/1471-2334-9-118

**Published:** 2009-07-28

**Authors:** Martina Saláková, Vratislav Němeček, Ruth Tachezy

**Affiliations:** 1 Department of Experimental Virology, Institute of Hematology and Blood Transfusion, Prague, Czech Republic; 2 National Reference Laboratory for Viral Hepatitis, National Institute of Public Health, Prague, Czech Republic

## Abstract

**Background:**

The female lower genital tract is a gateway for pathogens entering the host through the mucous membrane. One of the prevalent human viruses is Torque teno virus (TTV). The major reported routes of TTV transmission are fecal-oral and parenteral. Furthermore, other modes of transmission, e.g. sexual contact, are suggested. To investigate the sexual route of TTV transmission, cervical smears of healthy women and those with cervical lesions were screened for the presence of TTV DNA.

**Methods:**

TTV DNA was studied in cervical smears of 95 patients with cervical lesions and 55 healthy women. Paired serum samples were available from 55 and 42 women, respectively. All healthy women had normal cytology while 44 patients had histologically confirmed low-grade lesion (LGL) and 51 high-grade lesion (HGL). TTV DNA was detected with primers specific for the non-coding region. In 40 paired cervical smears and serum samples, the phylogenetic group of TTV isolates was determined. The presence of HPV DNA in cervical smears was detected by means of PCR with MY09/11 primers.

**Results:**

The prevalence of TTV DNA in cervical smears of healthy women was 52.7% and was comparable with that in paired serum samples (50%). Symptomatic women had significantly higher prevalence of TTV DNA in cervical smears (74.7%) than healthy controls. The TTV DNA prevalence in patient serum samples was 51%. The phylogenetic groups of TTV serum isolates were concordant with those of TTV from cervical smears of the same subjects. In cervical smears, a wider variety of TTV isolates was found. The viral loads in cervical smears were 10 to 1000 times as high as in sera. The HPV-positive study subjects had significantly higher TTV DNA prevalence than HPV negatives. The prevalence of TTV was not associated with disease severity.

**Conclusion:**

High prevalence of TTV in cervical smears suggests that sexual transmission is another mode of expansion of TTV infection among the population. The higher viral load in cervical smears than in the respective serum samples might indicate active TTV replication in the female genital tract. Nevertheless, cooperation between TTV and HPV needs to be further investigated.

## Background

The mucosal surface of the female lower genital tract is the first line of defense against pathogens. The physical barrier created by epithelial cells prevents the penetration of microbes and together with dendritic cells, macrophages, NK cells and chemical elements, they are the main components of the innate immune system. However, many common infectious disease pathogens succeed in entering the host through the mucous membrane after which they spread systematically. One of the most prevalent viral pathogens of cervical epithelial cells is human papillomavirus (HPV). HPV is a very common sexually transmitted infection with prevalence rates ranging from 2% to 44% among healthy women around the world [1]. HPV plays a role in the development of precancerous lesions and squamous cell carcinomas in the anogenital region. However, most individuals eliminate the HPV infection without any evidence of disease and only very few HPV-infected women develop invasive cervical cancer after a long period. The number of sexual partners, smoking, and infections by other sexually transmitted pathogens are factors which increase the risk of cervical cancer in HPV infected individuals [2].

The human population is frequently infected with Torque teno virus (TTV), a small, non-enveloped DNA virus with a single stranded circular genome, recently classified into a floating family called *Anellovirus *[3]. TTV shows a high degree of genetic variability: until now, more than 40 genotypes of TTV divided into 5 major phylogenetic groups have been identified in various clinical specimens from humans, including blood and cervical smears [4]. To date, no clinical manifestations have been unequivocally associated with TTV, classified into orphan viruses [5]. Nevertheless, the high genetic diversity of TTV implies the risk that one of TTV genotypes may have pathogenic potential.

The major routes of transmission are parenteral and fecal-oral. Since TTV DNA was detected in semen and cervical smears, sexual transmission has also been considered as a possible mode of infection [6-9]. A study has observed increase in TTV prevalence with increasing number of sexual partners among drug users with liver disease [10]; on the other hand, several studies have reported that in at-risk groups of prostitutes, the rate of TTV viremia was not significantly different from that in controls [11-13]. Therefore, the role of sexual transmission remains unclear.

To investigate the possibility of sexual transmission of TTV, cervical smears from healthy women and those with cervical lesions were screened for the presence of TTV DNA. For most study subjects, parallel serum samples were also available for comparison and TTV subtyping. Co-infection with TTV and HPV was investigated to support the hypothesis of the sexual route of TTV transmission.

## Methods

### Study population

Cervical smears and sera samples were obtained from the bank of the National Reference Laboratory for Papillomaviruses in Prague. All samples were collected during a prospective study of HPV in patients treated for cervical lesions in 1996–1997 [14]. Cervical smears from 95 patients (mean age 35 years, age range 18–71) were randomly selected to form two balanced groups of patients, one with low-grade and one with high-grade cervical lesions. The control group of 55 healthy women (mean age 32 years, age range 18–61) was selected as age matched to the patients group. Parallel serum samples were collected from 55 patients and 42 healthy women. For all study subjects, cytology data was available and for patients, histology data at enrollment was obtained. Furthermore, the questionnaire data on medical history, education, socioeconomic status, and number of lifetime sexual partners was accessible. All healthy women had normal cytology with no pathological, clinical or cytological findings while in the patient group, 44 and 51 subjects had histologically confirmed low-grade lesions (LGL) and high-grade lesions (HGL), respectively. No informed consent from patient was needed by course of law in the Czech Republic before 2000. The study in 1996–1997 was approved by the Ethical committee of the institution.

### DNA extraction

Cervical brushes were transported in tubes containing phosphate-buffered saline solution (PBS) with 5 mM ethylene diamine tetraacetic acid (EDTA), pH 8.0, and stored at +4°C. The tubes were vortexed and cells were collected by centrifugation at 1,000 rpm for 10 min. Forty microlitres of each cell pellet were digested in 100 μl of lysis buffer (50 mM Tris/HCL, pH 8.0, 1% Tween-20, 5 mM EDTA, pH 8.0) containing 100 μg/ml proteinase K (Fermentas, Hanover, MD) at 37°C overnight. Proteinase K was inactivated at 95°C for 10 minutes and the samples were stored at -20°C. DNA was extracted from 200 μl of serum by QIAamp Blood kit (QIAGEN Ltd., Crawley, UK) and dissolved in 100 μl of the elution buffer (QIAGEN Ltd., Crawley, UK). The control plasmid with a part of the human beta-globin gene was added to serum sample for the evaluation of DNA quality. Extracted DNA was stored at -20°C.

The integrity and quality of DNA was assessed by amplification of the human beta-globin gene with primer PC03/PC04. All tested samples were positive for the human beta-globin gene [15].

### HPV DNA detection

The cervical smears were analyzed for the presence of HPV DNA by means of PCR with MY09/11 primers followed by Southern blot hybridization and identification of HPV types on dot blot hybridization with oligonucleotide type specific probes or by sequencing [14].

### TTV DNA detection

TTV DNA detection in cervical smears and serum samples was done by nested PCR directed at the non-coding region of TTV (PCR NCR) with NG133/147 and NG132/134 [16] and with primers specific for the ORF1 N22 region (PCR ORF1) [17]. The sensitivity of both assays was analyzed by amplification of cloned TTV DNA at 1, 10 and 100 copies per tube. The PCR NCR system was 10–100 times more sensitive, able to detect one copy per reaction (data not shown). Furthermore, ORF system is known to amplify limited number of TTV genotypes while NCR system can detect genotypes from all five so far known major TTV phylogenetic groups. The prevalence with NCR primers in our study was much higher and therefore we used results obtained with this system for the final analysis.

Ten microlitres of the PCR product were separated electrophoretically on a 3% agarose gel (NuSieve 3:1, FMC BioProduct, Rockland, ME).

### Semiquantitative determination of TTV DNA load

DNA extracted from the sera and cervical smears from 5 patients with cervical abnormalities and 5 healthy women was serially diluted (in 10-fold steps) in distilled water and the presence of TTV DNA was detected by PCR NCR. The highest dilution (10^N^) testing positive was used as a relative titer for determining the viral titer per 1 ml of TTV DNA in serum samples and cervical smears.

### Genotyping determination

In 25 patients and 15 healthy women positive for TTV DNA in both cervical smears and serum samples, the TTV genotype was determined by a PCR assay based on amplification with TTV group-specific primers derived from the conserved non-coding region [18].

The statistical analysis was performed using the Fisher exact test. Odds ratios (OR) with 95% confidence intervals (CI) and two-tailed P values were calculated in 2 × 2 tables using the EPI INFO statistical package (2002 version) and Graph Pad InStat (version 3.05) (GraphPad Software, San Diego, CA). In all tests, the basic significance level was P = 0.05.

## Results

In total, 150 cervical smears and 97 serum samples were screened by PCR for the presence of TTV. The prevalence rates of TTV in the study groups of healthy women and patients with cervical lesions are shown in Table 1.

**Table 1:** Prevalence of TTV DNA in study groups

	Material				
	
Subjects	Cervical smears		Serum		OR (95%CI), P
	N	TTV positive (%)	N	TTV positive (%)	
Patients	95	71 (74.7)	55	28 (51)	2.9 (1.4–5.8), P = 0.004
Healthy women	55	29 (52.7)	42	21 (50)	NS

N – number of subjects, NS – not significant

The prevalence rates of TTV DNA were 52.7% in cervical smears of healthy women (29/55) and 50% in the parallel serum samples (21/42). The difference between the TTV DNA prevalence rates from the patient cervical smears and serum samples was statistically significant (74.7% (71/95) versus 51% (28/55), OR = 2.9, CI 1.4–5.8, P = 0.004). Comparable prevalence rates of TTV DNA were found in the sera from patients and healthy women, while patients showed significantly higher TTV DNA rates in cervical smears than healthy women (OR = 2.4, CI 1.2–4.8, P = 0.02).

Overall, 98.9% (94/95) of cervical smears from patients with cervical abnormalities and 27.3% (15/55) cervical smears from healthy women were HPV DNA positive. The HPV-positive study subjects showed a significantly higher prevalence of TTV DNA, i.e. 71.6% (78/109), than the HPV-negatives with 48.8% (20/41) (OR = 2.6, CI 1.3–5.5, P = 0.01). The patients were divided into two groups according to cervical lesion severity. The TTV prevalence rate was slightly higher in the group with high-grade lesions, i.e. 76.5% (39/51), than in that with low-grade lesions, i.e. 68.2% (30/44), but the difference was not statistically significant.

The relative viral loads in cervical smears and in the respective serum samples were compared for five patients and five healthy women. All of these subjects had 10 to 1.000 times higher TTV DNA titers in cervical smears than in serum samples.

TTV genotype was determined in 25 patients and 15 healthy women with both tested specimens positive for TTV. Any of TTV genotypes was recognized in 86% of patient cervical smears, 50% of patient serum samples, 77% of healthy women's cervical smears and 34% of the healthy women's serum samples. The most prevalent phylogenetic group was TTV 1 (45%), followed by TTV 3 (40%), the phylogenetic group TTV 5 was found rarely (6%) and TTV 2 only in the group of patients (4%). Concordant phylogenetic groups of TTV isolates were found in serum samples and the parallel cervical smears from the same subject; however, in cervical smears more diverse TTV genotypes were present (Table 2).

**Table 2:** TTV phylogenetic group distribution

ID	TTV phylogenetic groups in cervical smear	TTV phylogenetic groups in serum
1	3	3
2	1,3	3
3	1,3	1
4	1,2,3	3
5	1,3	1
6	1,2,3,5	1,2,3
7	1,3	1,3
8	1,3,5	1,3,5
9	1,3	1,3
10	1,3	1,3
11	1,3	1,3
12	3	3

TTV phylogenetic group distribution in parallel cervical smears and serum samples in which any TTV genotype was detected, ID is sample number, ID1-9 are samples from patients with cervical lesions, ID10-12 are samples from healthy women.

The number of sexual partners self-reported by the study subjects did not exceed four. No difference in the prevalence of TTV DNA associated with the number of sexual partners was found; the TTV DNA positivity rates were 65% (73/112) in subjects with less than three sexual partners and 64.5% (20/31) in those with three or four sexual partners.

## Discussion

TTV infects the majority of the general population worldwide but is not associated with any clinical evidence of disease. The high prevalence of TTV in the general population might be explained by the existence of multiple transmission routes. Several studies have reported the parenteral and fecal-oral routes of TTV transmission and other modes of transmission have also been considered. The presence of TTV DNA in cervical smears and semen suggests that TTV may be spread by sexual contact [9].

In our study, in agreement with Calcaterra et al., Chan et al., and Fornai et al [6-8], we observed a high prevalence of TTV in cervical smears. The prevalence of TTV DNA in cervical smears of healthy women was similar to that previously reported for the Czech general population [13]. In healthy women, no difference was found in the prevalence of TTV between the cervical smears and parallel serum samples. Only one study has so far compared the prevalence of TTV DNA in these two types of specimens. Fornai et al. have reported a higher prevalence of TTV DNA in serum than in cervical smears. Nevertheless, the differences between the study populations can explain this discrepancy. The subjects tested in the Fornai study were HIV-infected individuals who have a higher TTV prevalence and viral load in the serum than the general population [19,20].

The cervical smears from patients with cervical lesions were more frequently infected by TTV in comparison with those from healthy women. The co-presence of HPV and TTV in the patient cervical smears suggests that both viruses have a common transmission route. The co-infection with TTV and other viruses such as HBV, HCV, and HIV l has been reported by others [11,19,21]. The high TTV/HPV co-infection rate, similar to those of HIV, HBV, HCV, supports the hypothesis that these viruses share the same mode of transmission and that sexual transmission is another important mode of TTV infection. HPV is an etiological factor of cervical carcinoma and precancerous cervical lesions. The mode of transmission is mostly sexual. It has been documented that patients with cervical disorders are more likely to start sexual life sooner and to have more sexual partners [22]. Therefore, it is likely that if TTV is transmitted sexually it will be more frequently present in women infected with HPV. Women with cervical lesions are also more likely to be HPV positive and form a risk group in comparison to healthy women in terms of the mode of transmission. Nevertheless, we did not find the number of sexual partners to be a risk factor for TTV infection, most likely due to the relatively low number of lifetime sexual partners of our study subjects. Many studies have reported that the risk of HPV infection and cervical cancer increases with more than six lifetime partners [22]. The similarity in the TTV phylogenetic groups in the isolates from cervical smears and serum samples implies the same source of TTV infection. The higher TTV load in cervical smears may indicate that the vagina is the entry site of TTV infection. The higher genotypic variability of TTV in cervical smears may reflect that the vagina is the first place of TTV infection. On the other hand, it might also explain the inability to detect the lower TTV load in the serum.

Originally, liver tissue had been thought to be a site of TTV replication since the viral loads in hepatocytes were 10 to 100 times higher than those in the respective plasma samples. This hypothesis was confirmed by the detection of a double-stranded variant of TTV DNA in the liver and hepatocytes by in situ hybridization. The viral titer was 10 to 10.000 times higher in saliva than in serum. The assumption that TTV might replicate in oropharyngeal tissues and/or salivary glands was supported by the detection of TTV in the gingival tissue [23]. In our study, we found 10 to 1.000 higher viral loads in cervical smears than in the parallel serum samples. This observation suggests that TTV might also replicate in cells of the cervicovaginal area.

The reported higher viral loads of TTV in HIV infected patients might be associated with a poorer prognosis in AIDS. A higher viral load can be explained by the immune stimulation due to HIV infection. CIN biopsies in HIV-positive women have shown higher lymphocyte and macrophage counts in the squamous epithelium of the cervix and an increased intralesional concentration of proinflammatory cytokines modulating HIV replication, with consequently increased HIV genital shedding [24]. Similarly, higher TTV prevalence in HPV-positive patients can be attributed to the stimulation of the immune system by HPV infection and additional increase in TTV load. Nevertheless, more data is needed to better understand the phenomena. Additional risk to patients with cervical lesions from co-infection with TTV was also investigated. As in our study, TTV prevalence did not differ between patients with low-grade and high-grade cervical lesions, co-infection with TTV and HPV seemed unlikely to promote the progression of precancerous cervical lesions.

## Conclusion

To conclude, high TTV prevalence in cervical smears suggests that sexual transmission is another mode of expansion of TTV infection among the population. The higher viral loads in cervical smears than in the respective serum samples might indicate active TTV replication in the female genital tract or might be the result of plasma secretion. The TTV shedding is possibly mediated by the immune upregulation in the cervix and vagina due to HPV infection. Nevertheless, natural cooperation between TTV and HPV needs to be further investigated.

## Competing interests

The authors declare that they have no competing interests.

## Authors' contributions

MS projected the study, carried out the experimental work, did all statistical analyses and evaluation of the results. MS wrote the first draft of the paper and other coauthors contributed to the final draft. RT and VN participated in the study design and data interpretation. All authors read and approved the final manuscript.

## Pre-publication history

The pre-publication history for this paper can be accessed here:

http://www.biomedcentral.com/1471-2334/9/118/prepub
